# Mixed Effects Modeling of Proliferation Rates in Cell-Based Models: Consequence for Pharmacogenomics and Cancer

**DOI:** 10.1371/journal.pgen.1002525

**Published:** 2012-02-09

**Authors:** Hae Kyung Im, Eric R. Gamazon, Amy L. Stark, R. Stephanie Huang, Nancy J. Cox, M. Eileen Dolan

**Affiliations:** 1 Department of Health Studies, University of Chicago, Chicago, Illinois, United States of America; 2 Department of Medicine, University of Chicago, Chicago, Illinois, United States of America; 3 Department of Human Genetics, University of Chicago, Chicago, Illinois, United States of America; University of Washington, United States of America

## Abstract

The International HapMap project has made publicly available extensive genotypic data on a number of lymphoblastoid cell lines (LCLs). Building on this resource, many research groups have generated a large amount of phenotypic data on these cell lines to facilitate genetic studies of disease risk or drug response. However, one problem that may reduce the usefulness of these resources is the biological noise inherent to cellular phenotypes. We developed a novel method, termed Mixed Effects Model Averaging (MEM), which pools data from multiple sources and generates an intrinsic cellular growth rate phenotype. This intrinsic growth rate was estimated for each of over 500 HapMap cell lines. We then examined the association of this intrinsic growth rate with gene expression levels and found that almost 30% (2,967 out of 10,748) of the genes tested were significant with FDR less than 10%. We probed further to demonstrate evidence of a genetic effect on intrinsic growth rate by determining a significant enrichment in growth-associated genes among genes targeted by top growth-associated SNPs (as eQTLs). The estimated intrinsic growth rate as well as the strength of the association with genetic variants and gene expression traits are made publicly available through a cell-based pharmacogenomics database, PACdb. This resource should enable researchers to explore the mediating effects of proliferation rate on other phenotypes.

## Author Summary

Cell-based models provide a convenient system to conduct studies that would be impossible to apply to human subjects, but the phenotypes measured on these models can be marred with biological noise. We propose a method (MEM) to address this issue by statistically combining data from various sources, and we apply it to the proliferation rates of cell lines collected as part of the International HapMap project. We show that the proliferation rate computed using our method is a better measure of the true proliferation rate of the cells and produces a much stronger association with gene expression phenotypes on the same cell lines: more than 30% of the genes tested were significantly associated with proliferation rate. We also demonstrate that genetic variants have an effect on growth rate. Finally, we make these intrinsic proliferation rates and the strength of the association with gene expression phenotypes public, which should allow other researchers to explore the mediating effects of proliferation on other phenotypes.

## Introduction

The International HapMap project [1], [2] has made available a vast amount of genetic variation data from a large number of individuals with diverse ethnic background. A recent population based whole-genome sequencing initiative (1000 Genomes Project [3]) sought to expand on this effort by providing a more comprehensive catalog of human genome sequence variation, including rare variants in these samples. These data can be used to study the effect of genetic variants on disease processes, pharmacologic traits, and environmental responses. As part of the HapMap project, EBV-transformed lymphoblastoid cell lines (LCLs) derived from individuals of diverse ancestry were established, which provide renewable sources of DNA and RNA. The commercial availability of these cell lines and the rich genetic information publicly available have enabled a large number of researchers to adopt them as *in vitro* models for the study of genotype-phenotype relationships in human cells [4]. Consistent with this trend, a vast amount of phenotypic data such as gene expression levels, drug response, and radiation response have been made publicly available [5]–[8]. Furthermore, an enormous amount of genotype-phenotype relationships have been generated [4], [9]–[11]. Our group has therefore constructed a database, PACdb [6], a public central repository of pharmacology-related phenotypes, to host these integrative results obtained in HapMap LCLs.

Although there are many advantages in utilizing the cell-based system for genotype-phenotype studies, the problem of biological and experimental noise when dealing with LCL-based phenotypes and the potential for spurious results has been recognized by several researchers [12], [13]. Indeed, it has been proposed [14] that non-genetic confounders and other technical factors in generating phenotypes from these cell lines may hamper efforts to evaluate the genetic contributions to phenotype. One common factor, cellular growth rate, has undergone scrutiny for its effect on various phenotypes, particularly drug-induced cytotoxicity, a phenotype of interest in pharmacogenomic studies [12], [13]. For example, aberrant growth rate is one of the distinctive features of cancer cells and growth inhibition following exposure to chemotherapeutics and other cytotoxics is intimately related to growth rate [13]. Thus, studying cellular proliferation rate is likely to advance our understanding of cancer pathogenesis. In this study, we set out to extract and combine data from various sources and calculate intrinsic cellular growth rate using a novel mixed effects model (MEM) for over 500 HapMap cell lines.

Previous studies have shown the presence of a strong correlation between gene expression traits and growth rate in other organisms such as yeast and bacteria [15]–[17]. Brauer et al. [15] measured gene expression traits in yeast under several controlled growth conditions and reported that 25% of the gene expression phenotypes were correlated with growth rate. In addition, genes important for cellular proliferation have been found to be differentially expressed in most cancer tissues [18]–[20]. Such genes were shown to be strong prognostic factors in breast cancer [21]–[23], renal cancer [21], lung cancer [21], mantle cell lymphoma patients [24]. Thus, to gain insights into the factors contributing to intrinsic growth rate phenotype, we also evaluated the relationship between gene expression and cellular growth.

## Results

Our laboratory has assayed over 500 HapMap LCLs for drug-induced cellular sensitivity phenotypes for a wide spectrum of chemotherapeutic agents and investigated the genetic variants and genes that affect drug response [25], [26]. This set comprises the 180 Utah residents of Western European ancestry (CEU), 180 African from Ibaban, Nigeria (YRI), 90 Asian (ASN, composed of 45 Han Chinese from Beijing and 45 Janapase from Tokyo), and 90 African American from the Southwestern US (ASW) cell lines. For more than 10 chemotherapeutic drugs, cellular growth inhibition after exposure to a range of concentrations of the drug was measured using the alamarBlue assay as described previously [12]. For every single drug sensitivity experiment, the cellular growth rate *without* drug was also determined, which provided us with a large number of replicated measures of cellular growth rate under a wide range of biological conditions (e.g., freeze and thaw, passage of cells, personnel performing experiments). For example for 90 of CEU phase I and II and 90 of YRI phase I and II LCLs, we had 10 different measurements performed over the course of several years (2006–2011).

### Intrinsic growth rate

**Figure 1:**
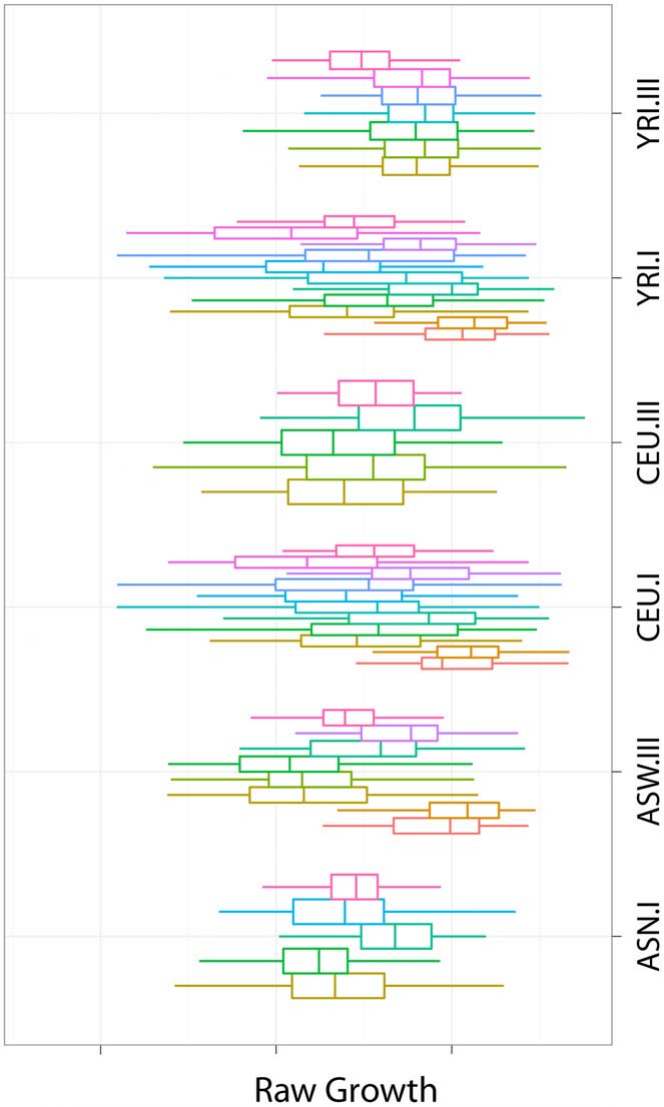
Growth rate measurements by HapMap panel and experiment. We illustrate the intrinsic growth rate computed with MEM in comparison with the raw data. The rightmost boxplot in each panel (label 12) represents the intrinsic growth rate. The other boxplots correspond to the raw data. The variability of the raw data is in general much larger than that of the intrinsic growth.

We have computed the intrinsic growth rate of these cell lines using MEM (described in Materials and Methods section) and provide the values in Table S1. Figure 1 shows these values in comparison with the raw data. The rightmost boxplot in each panel (label 12) represents the intrinsic growth rate. The other boxplots correspond to the raw data. It is clear that the variability of the raw data is in general much larger than the intrinsic growth. Unless otherwise stated, all subsequent analyses are done on the intrinsic growth rate.

### Population and gender differences exist in LCL intrinsic growth rate

**Figure 2:**
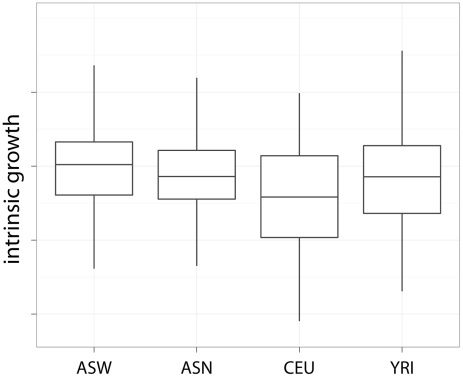
Intrinsic growth rate by population. This plot shows the intrinsic growth rate as a function of population. The fastest growing population was ASW, followed by YRI and ASN with roughly similar growth rate, and lastly CEU.

The mean and standard deviation of the intrinsic growth by population is shown in Table 1. The effect of population on growth rate was significant (likelihood ratio test 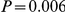) with ASW growing the fastest, followed by YRI and ASN, and CEU being the slowest. In pairwise comparison, only CEU's growth was significantly (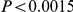) lower than ASW's. Similar results had been reported for YRI, ASN and CEU by Stark et al. [13] but the wide range of experimental conditions used in the current study strengthens the evidence. Figure 2 shows boxplots of intrinsic growth rate by population. Since we have reduced the effect of confounders by combining data from multiple experiments, the differences we find are likely to be intrinsic to the cell lines. However, the generalizability of these results to in vivo population differences is not obvious. One factor that may explain the slow growth of CEU cell lines is the time from the establishment of these lines and merits further study.

**Table 1:** Average intrinsic growth rate by population and gender.

Population	mean	sd
ASW	79302	11776
ASN	76484	11275
CEU	71724	13552
YRI	76970	14188

Gender	mean	sd
Male	77578	13425
Female	73520	13086

This table shows the mean and standard deviations of the intrinsic growth rate computed using MEM by population and gender.

**Figure 3:**
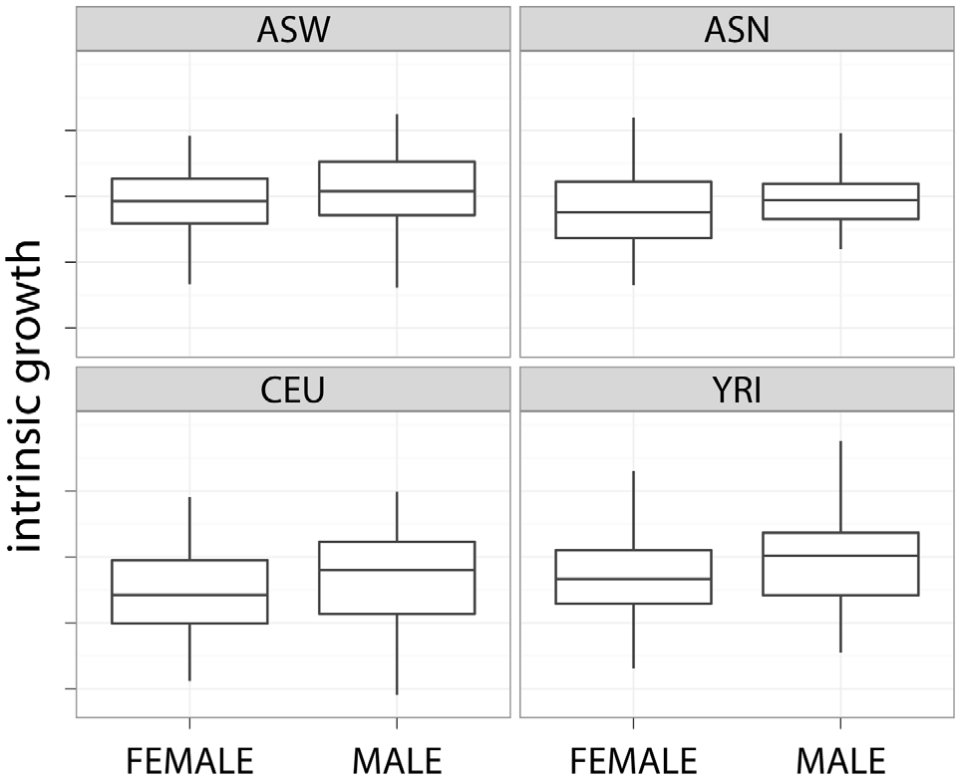
Slower growth for female cell lines. This plot shows the slower growth of female cell lines across different populations.

The mean and standard deviation of the intrinsic growth rate by gender are also shown in Table 1. Figure 3 shows the boxplot of the latter by gender and population. The effect of gender on growth rate was found to be significant. Across all experiments, female cell lines grew approximately 7% (95% CI) slower than male cell lines. This finding remained quite consistent across individual experiments. Gender differences are not likely to be due to experimental differences since both female and male cell lines are handled similarly. Differential effect of estrogen and other hormones in media could explain part of this difference.

### Comparison with other publicly available LCL growth rate dataset

Choy et al. [14] measured growth rate on some of the same cell lines and made the data publicly available (CEU I/II, YRI I/II, and ASN). They performed cell counting for five consecutive days and estimated growth rate as the slope of the fitted line (log concentration vs. time). This growth rate is based on only one biological replicate (although they did measure an additional biological replicate at one time point for a subset of cell lines for internal validation). We compared our estimated intrinsic growth rate with the growth rate measured by Choy et al. [14]. The correlation between Choy's growth rate and ours was 0.30, which supports the idea that both our intrinsic growth and Choy's growth are realizations of the true intrinsic growth rate albeit with different degree of noise. We analyzed Choy's data and found comparable magnitude of the effect of gender (slightly less than 4%; p = 0.07) and cell lines from CEU population grew at the slowest pace in both datasets.

### Cellular growth rate is important for gene expression

**Figure 4:**
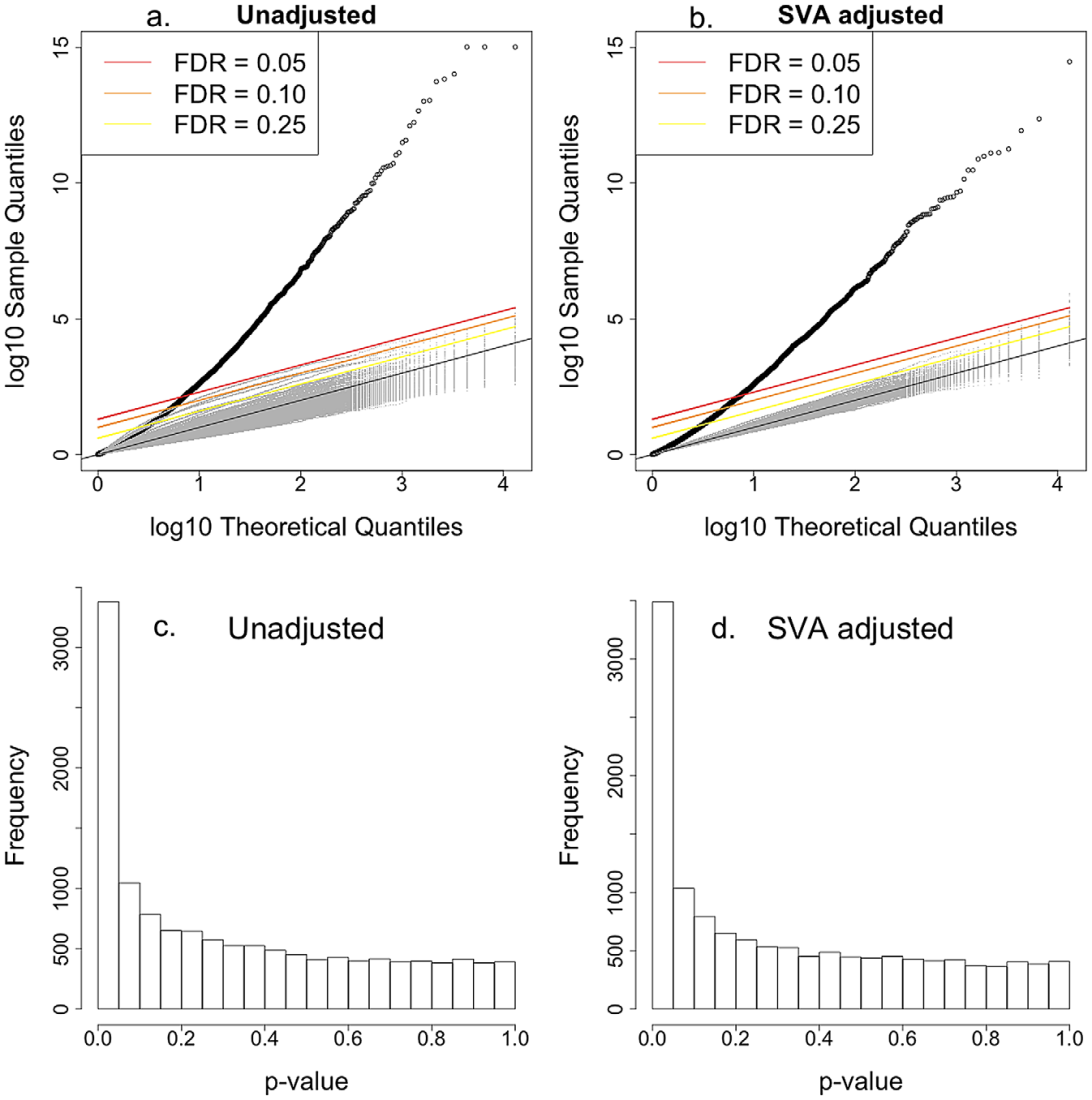
Intrinsic growth versus gene expression with Surrogate Variable Analysis adjustment. a. QQ-plot of the association p-values between gene expression and intrinsic growth adjusted by gender and population. b. QQ-plot of the association p-values between gene expression and intrinsic growth adjusted by gender, population, and expression heterogeneity. Points above the red, orange, and yellow lines have FDR less than 0.05, 0.10, and 0.25 under the assumption of no correlation between gene expressions. Gray line is the one to one line. Gray dots correspond to 200 associations estimated after permuting the growth rate so the null hypothesis of no association would hold. It is clear that the high significance of p-values is not due to model misspecification nor correlations between gene expression levels. c. Histogram of association p-values from a. d. Histogram of association p-values from b.

Baseline gene expression data for CEU and YRI phase I and II described in Zhang et al. [27] were used to examine the association with the intrinsic growth rate. We found that almost 3000 out of the 10748 genes examined were associated with the intrinsic growth rate at FDR
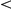
0.10; the result held regardless of whether we adjusted for expression heterogeneity or not [28]. The list of genes whose expression level associated with intrinsic growth rate in CEU and YRI LCLs is provided in Table S2. Figure 4 shows the QQ-plot of p-values from the association between gene expression phenotypes and intrinsic growth rate adjusted by gender and population. The upper left panel shows the QQ-plot for the unadjusted analysis and the upper right panel shows the plot for the Surrogate Variable Analysis (SVA; expression heterogeneity) adjusted analysis. The lower panels show the corresponding histograms with p-values highly concentrated near the zero. The gray dots in each of the QQ-plots correspond to p-values under the null hypothesis of no association, which was computed by randomly permuting the phenotype. Actual p-values lie well above the null hypothesis p-values, which indicate that the significance found is not due to model misspecification or correlation between gene expression phenotypes. Stark et al. [13] had not been able to find significant association (FDR
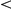
0.10) between gene expression and growth rate in the first phase CEU population because of the noisier version of growth rate and smaller sample size used at the time. The strong correlation between gene expression traits and cellular growth rate is consistent with similar findings in yeast [15] and in bacteria [16], [17].

**Figure 5:**
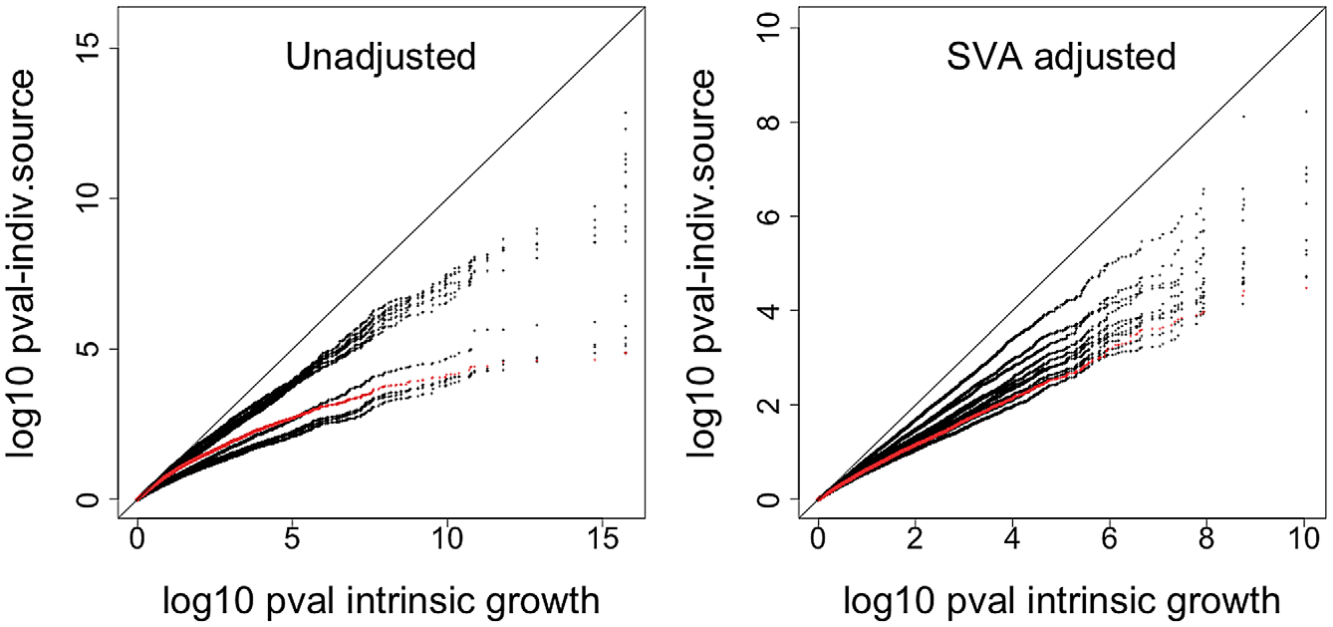
Comparison of significance between intrinsic growth and individual experiment's growth. The plot shows the QQ-plot comparing the p-values from the association between gene expression phenotype and growth rate when the intrinsic value computed with MEM is used vs. when the individual experiment's values are used. The left panel shows the p-values from the SVA-adjusted analysis and the right panel shows the results from the unadjusted analysis. All points are below the one to one line, which means that the intrinsic growth rate achieves greater power in identifying association than any of the individual experiment's data. The red line corresponds to growth rate data from Choy et al. [14].

A clear advantage of using our method is shown by the fact that our power to detect association between gene expression phenotype and growth rate is substantially increased. This is illustrated in Figure 5, which compares the p-values from the association between gene expression phenotype and growth rate when the intrinsic value computed with MEM is used vs. when the individual experiment's values are used. The left panel shows the p-values from the SVA (expression heterogeneity) adjusted analysis and the right panel shows the results from the unadjusted analysis. All points lie below the one-to-one line, which means that the intrinsic growth rate achieves greater power in identifying association than any of the individual experiment's data. The red dots correspond to growth rate data from Choy et al. [14].

### Functional significance of growth-rate associated genes

Functional enrichment analysis was performed using DAVID Bioinformatics Resources [29], [30]. Table 2 shows the GO terms that were enriched in our intrinsic growth rate-associated gene set. They clustered into cell cycle, cell death, intracellular transport, protein transport and phosphorylation. For comparison, we checked the proportion of cell cycle, mitosis, cell death and phosphorylation genes among metabolic process genes (from GO) and immune response genes (from GO). The proportion of growth-associated genes annotated to these terms were 6.8%, 2.7%, 6.1%, and 6.2%, respectively. In comparison, among immune response genes 0.2%, 0.16%, 1.1%, and 0% were annotated to these terms. For metabolic process genes none of these terms reached significance at the loose threshold of 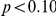.

**Table 2:** GO enrichment analysis of growth-associated genes.

Term	Count	%	PValue	Bonferroni
GO:0007049 cell cycle	190	6.80	3.09E-11	1.35E-07
GO:0007067 mitosis	74	2.65	4.79E-11	2.08E-07
GO:0000280 nuclear division	74	2.65	4.79E-11	2.08E-07
GO:0048285 organelle fission	76	2.72	5.26E-11	2.29E-07
GO:0000087 M phase of mitotic cell cycle	74	2.65	1.23E-10	5.35E-07
GO:0000278 mitotic cell cycle	105	3.76	3.25E-10	1.42E-06
GO:0033554 cellular response to stress	144	5.15	6.78E-10	2.95E-06
GO:0022402 cell cycle process	142	5.08	2.13E-09	9.28E-06
GO:0016265 death	170	6.08	1.08E-08	4.68E-05
GO:0008219 cell death	169	6.05	1.10E-08	4.80E-05
GO:0046907 intracellular transport	157	5.62	1.22E-08	5.32E-05
GO:0012501 programmed cell death	145	5.19	7.46E-08	3.24E-04
GO:0051301 cell division	81	2.90	1.93E-07	8.37E-04
GO:0022403 cell cycle phase	105	3.76	2.06E-07	8.97E-04
GO:0006796 phosphate metabolic process	210	7.51	2.15E-07	9.35E-04
GO:0006793 phosphorus metabolic process	210	7.51	2.15E-07	9.35E-04
GO:0006915 apoptosis	141	5.04	2.62E-07	1.14E-03
GO:0000279 M phase	87	3.11	3.87E-07	1.68E-03
GO:0009057 macromolecule catabolic process	172	6.15	8.49E-07	3.68E-03
GO:0043067 regulation of programmed cell death	177	6.33	1.16E-06	5.04E-03
GO:0010941 regulation of cell death	177	6.33	1.48E-06	6.41E-03
GO:0015031 protein transport	167	5.97	1.72E-06	7.43E-03
GO:0045184 establishment of protein localization	168	6.01	1.97E-06	8.54E-03
GO:0006974 response to DNA damage stimulus	93	3.33	2.39E-06	1.03E-02
GO:0016310 phosphorylation	173	6.19	2.64E-06	1.14E-02
GO:0044265 cellular macromolecule catabolic process	159	5.69	3.00E-06	1.30E-02
GO:0007346 regulation of mitotic cell cycle	47	1.68	3.31E-06	1.43E-02
GO:0042981 regulation of apoptosis	173	6.19	3.56E-06	1.54E-02
GO:0030163 protein catabolic process	137	4.90	1.28E-05	5.43E-02
GO:0007088 regulation of mitosis	23	0.82	1.46E-05	6.15E-02

This table shows the top GO terms that were enriched in our growth-associated gene set. They clustered into cell cycle, cell death, intracellular transport, protein transport and phosphorylation. It was obtained using the DAVID Bioinformatic Resources.

Table 3 shows the SP-PIR keywords enriched in our growth gene set; more than half of the growth-associated genes were associated with phosphoprotein (p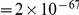) and 22% of them were associated with acetylation (p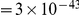). For comparison, we checked the proportion of genes related to phosphoprotein keyword for two other cellular functions: metabolism (metabolic process from GO) and immune response. None of these genes were annotated with the phosphoprotein keyword in the SP-PIR database.

**Table 3:** SP-PIR keyword enrichment of growth-associated genes.

Term	Count	%	PValue	Bonferroni	Benjamini
phosphoprotein	1465	52.42	3.25E-70	2.31E-67	2.31E-67
acetylation	630	22.54	9.54E-46	6.78E-43	3.39E-43
nucleus	823	29.45	4.49E-24	3.19E-21	1.06E-21
cytoplasm	653	23.36	1.43E-20	1.02E-17	2.54E-18
atp-binding	303	10.84	4.29E-18	3.05E-15	6.10E-16
nucleotide-binding	356	12.74	3.00E-15	2.13E-12	3.55E-13
transferase	304	10.88	6.20E-15	4.42E-12	6.31E-13
alternative splicing	1255	44.90	2.82E-14	2.00E-11	2.51E-12
cell cycle	127	4.54	1.49E-13	1.06E-10	1.18E-11
kinase	168	6.01	1.35E-12	9.62E-10	9.62E-11
host-virus interaction	87	3.11	4.10E-12	2.91E-09	2.65E-10
endoplasmic reticulum	167	5.97	5.71E-11	4.06E-08	3.38E-09
ubl conjugation	140	5.01	7.40E-10	5.26E-07	4.05E-08
mitosis	59	2.11	1.63E-09	1.16E-06	8.29E-08
cell division	76	2.72	2.00E-09	1.42E-06	9.48E-08
ligase	84	3.01	2.68E-09	1.91E-06	1.19E-07
golgi apparatus	135	4.83	1.70E-08	1.21E-05	7.12E-07
Apoptosis	94	3.36	9.98E-08	7.09E-05	3.94E-06
ATP	63	2.25	9.92E-07	7.05E-04	3.71E-05
ubl conjugation pathway	113	4.04	1.57E-06	1.12E-03	5.59E-05
phosphotransferase	55	1.97	2.33E-06	1.66E-03	7.89E-05
lysosome	44	1.57	3.35E-06	2.38E-03	1.08E-04
serine/threonine-protein kinase	88	3.15	4.86E-06	3.45E-03	1.50E-04
Aminoacyl-tRNA synthetase	18	0.64	9.49E-06	6.72E-03	2.81E-04
protein transport	104	3.72	1.99E-05	1.41E-02	5.67E-04
activator	109	3.90	3.42E-05	2.40E-02	9.34E-04
mitochondrion	161	5.76	4.49E-05	3.14E-02	1.18E-03
helicase	38	1.36	4.54E-05	3.18E-02	1.15E-03
rna-binding	111	3.97	6.66E-05	4.62E-02	1.63E-03
wd repeat	64	2.29	9.59E-05	6.59E-02	2.27E-03
transit peptide	99	3.54	1.09E-04	7.47E-02	2.50E-03
phospholipid biosynthesis	17	0.61	1.25E-04	8.48E-02	2.76E-03
Chromosome partition	14	0.50	1.72E-04	1.15E-01	3.71E-03
transcription factor	22	0.79	1.88E-04	1.25E-01	3.92E-03
hydrolase	272	9.73	2.19E-04	1.44E-01	4.45E-03
cytoskeleton	124	4.44	2.63E-04	1.70E-01	5.18E-03
isopeptide bond	69	2.47	4.33E-04	2.65E-01	8.29E-03
endosome	49	1.75	5.30E-04	3.14E-01	9.87E-03

This table shows the top SP-PIR keywords enriched in our growth-associated gene set. More than half of the growth-associated genes were associated with phosphoprotein (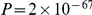) and 22% of them were associated with acetylation (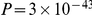). It was obtained using the DAVID Bioinformatic Resources.

Table S3 shows the Kegg pathways enriched in our set.

### Cell proliferation signatures

**Figure 6:**
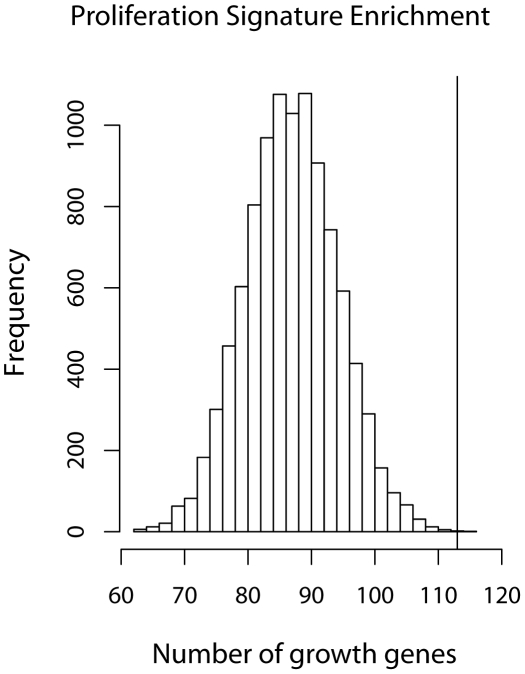
Growth gene overlap with Wirapati et al's proliferation signature. Histogram of the number of growth genes (as defined by 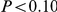) when a random set of 235 genes were sampled out of 10748 genes. The maximum number of growth genes was 101 after 10000 simulations. The black circle indicates the 104 growth genes found in Wirapati's proliferation signature [31].

We compared the growth-associated genes with two recently published proliferation signatures. The first one was obtained by performing a meta-analysis of over 2833 breast tumor expression profiles by Wirapati et al. [31]. The second one was compiled by Starmans et al. [21] based on cell cycle in cervix cancer cell lines [32] and human fibroblasts [33]. We found that 44% of Wirapati's proliferation genes belonged [31] to our growth-associated gene list (defined as 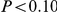) and 75% of them had a positive effect on growth (higher expression associated with faster growth). This enrichment is not likely to occur by chance as can be seen in Figure 6. The figure shows a histogram of the number of growth-associated genes we would get if we randomly sampled the set of all genes we considered. The vertical line indicates the actual number of growth-associated genes in Wirapati's list. We performed the same analysis with Starmans et al. [21] proliferation signature but did not find any significant enrichment. Nevertheless, 80% of the Starmans' proliferation genes had a positive effect on LCL growth rate.

### Enrichment of growth-associated gene eQTLs among top growth-associated SNPs

We performed a genome wide association study (GWAS) of the intrinsic growth rate for CEU and YRI unrelated cell lines. Even though we were unable to find genome-wide significant SNPs, we did find that the top intrinsic growth-associated SNPs were more likely to target (as eQTLs) intrinsic growth-associated genes. We have quantified the enrichment using three different procedures described in the Methods section. The first one uses the hypergeometric distribution to test the enrichment of growth associated genes among targets of growth associated SNPs. The Fisher's test p-value was 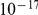. The second method accounts for the correlation between genes and yielded an empirical 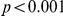 (none of the 1000 simulations yielded Fisher's test p-value smaller than the observed 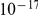). The third method accounts for the fact that the data from the same individuals are used when computing the intrinsic growth-gene association as well as the intrinsic growth-SNP association. The empirical p-value with this method was 0.026.

### Growth-associated gene eQTLs

Since eQTLs have been shown to be more likely to be associated with complex phenotypes [34]–[37], we focused the analysis on growth-associated gene eQTLs but the enrichment was not strong enough to render significant SNPs after adjusting for multiple testing. Interestingly however, among the growth-gene eQTLs we found two well-replicated colorectal cancer SNPs: rs4779584 [38], [39] and rs3802842 [39], [40]. A target gene of rs4779584 is a growth-associated gene *NEU1* [MIM:608272] (growth gene expression association 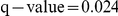), which has been reported to contribute to the suppression of metastasis of human colon cancer [41]. A target gene of rs3802842, *MED13* [MIM: 603808] (growth gene association q-value = 0.052), is part of the CDK8 subcomplex [42] and CDK8 is a colorectal oncogene that regulates beta catenin activity [43]. To our knowledge, the potential functional connection between these two established colorectal cancer SNPs and the growth-associated genes *NEU1* and *MED13* has not been made previously. The association between these SNPs and intrinsic growth rate however was not significant.

### Effect of EBV copy number on intrinsic growth accounted by SVA

As an attempt to address the concern of whether these associations may be confounded by EBV transformation, we cross-checked the growth-associated genes with the list of EBV transformation-associated genes reported by Caliskan et al. [44] and found no evidence of enrichment of EBV genes among our growth genes. Furthermore, we analyzed the effect of EBV copy numbers on intrinsic growth. For this purpose, we used measurements of EBV copy numbers on a large portion of cells used for the association with gene expression (86 CEU, 74 YRI) from Choy et al. [14]. We found a small but significant effect (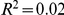) of EBV on intrinsic growth. However, once we accounted for SVA variables (expression heterogeneity), the effect was no longer significant. Similar results were found using EBV data generated in our lab for a subset of the samples. This result suggests that the intrinsic growth-gene expression associations we found (SVA adjusted) are not mediated by EBV copy numbers, consistent with the lack of enrichment in EBV-related genes among our top growth genes.

## Discussion

In this study, we propose a novel method, MEM, that combines data from multiple sources using a mixed effects model and estimates an intrinsic phenotype that is more reflective of the true phenotype than each of the individual experiment's data. We apply it to generate intrinsic cellular growth rate, which is a phenotype with important implications for disease biology and phamacogenomics. Using MEM we computed the intrinsic growth rate of over 500 HapMap cell lines and studied their properties. To our knowledge, this is the most comprehensive analysis to date of intrinsic cellular growth rate for the HapMap cell lines, for which various biological conditions were included in the estimation. We understand that estimates of intrinsic growth can be further improved as more experiments are included. A Bayesian approach would fit well for this purpose. Existing data would make up the prior distribution for the intrinsic growth rates and the addition of new data would generate posterior distributions, presumably more concentrated on the true intrinsic growth rates. We plan to regularly update the HapMap LCL intrinsic growth rate phenotype data and make them widely available to the research community through PACdb [6].

We found significant *in vitro* population differences in cellular growth rate in the HapMap populations included in our study. The ASW lines (African American) proliferated at the fastest rate followed by YRI and ASN (Asian), and CEU were the slowest. We analyzed Choy et al.'s [14] growth rate data and also found CEU lines to grow slower than other populations. Since we combined data from multiple sources and reduced the level of noise, the observed population differences are likely to be intrinsic to the cell lines and may in part be due to genetic factors; however, the methods used in establishing the LCLs and the experimental conditions during the EBV-transformation could also contribute to this observation. The fact that CEU cell lines were established much earlier than other populations could in part explain their slow growth. Of the populations included in the HapMap Project, only the CEU LCLs existed as previously established cell lines. The other populations were collected and established as cell lines specifically for the HapMap Project over the years 2002 through 2007 [12], [13]. Nonetheless, the observed population difference in intrinsic cellular growth rate needs to be considered when studying population differences in complex traits using these cell lines.

Interestingly, we found that female cell lines grow at roughly 7% slower pace than male cell lines consistently across different experiments. We found similar gender effect when we analyzed Choy et al.'s data [14]. Gender differences are not likely to be due to experimental differences since both female and male cell lines are handled similarly. Differential effect of estrogen and other hormones in media could explain part of this difference.

It is not clear whether these observed population and gender differences are extensible beyond these cell lines. However, these initial observations warrant further studies.

We found that almost 3000 gene expression phenotypes were associated with the intrinsic growth rate, which is consistent with findings in yeast [15]. This finding held robustly, whether or not we accounted for expression heterogeneity using Surrogate Variable Analysis [28]. Our top growth-related genes were enriched in cell cycle, mitosis, cell death, and phosphorylation terms. We also found a significant overlap between our intrinsic growth genes and a proliferation signature inferred from breast tumor microarray data [31]. Thus, our study provides a comprehensive list - combining both germline and tumor cells - of potential biomarkers and therapeutic targets for proliferation-mediated phenotypes. Furthermore, the gene expression traits associated with intrinsic growth determined by our study are much more significant than their corresponding associations with growth rate data generated from any individual experiment, including Choy et al.'s [14] growth rate. This strongly suggests that our method to combine data from several experiments is succeeding at yielding a more intrinsic measure of growth rate.

Despite the limited power given the relatively small sample size used for eQTL mapping, we demonstrate evidence of genetic effect on intrinsic growth rate by determining the enrichment of growth-associated genes among genes targeted by top growth-associated SNPs (as eQTLs) after accounting for LD structure and correlation between gene expressions. Interestingly, among intrinsic growth gene eQTLs, we found two well replicated colorectal cancer SNPs (rs4779584 [38], [39] and rs3802842 [39], [40]), which target growth-associated genes *NEU1* and *MED13*; both genes have been implicated in colorectal cancer [41]–[43].

We deposited our findings into PACdb [6], which should be a useful addition to the already rich set of phenotype data currently available for the HapMap cell lines. In addition to the intrinsic growth rates (Table S1), the significance of the association with gene expression phenotypes (Table S2) is available from the same database. This resource should be useful to explore any mediation effect of growth rate on the phenotype of interest either by using the intrinsic growth rate as a covariate in the analysis or by looking at overlap between the phenotype of interest and the top growth related genes. We also make the R code to apply MEM and generate intrinsic growth available on PACdb (http://pacdb.org/growthrate/generate-igrowth.r and http://pacdb.org/growthrate/rawgrowth.txt)

## Materials and Methods

### Mixed Effects Model averaging MEM

MEM pools phenotype data from multiple sources and computes an intrinsic value of the phenotype for each individual after accounting for different experimental conditions and covariates.
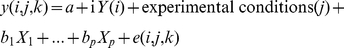
where the index 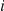 identifies the individual or cell line, i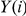 represents the intrinsic phenotype of the individual, experimental conditions can represent a large range of different experimental conditions (for example different sites, technicians, method, passage number, etc.), 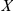
's are relevant covariates, and 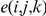 is an error term. The index 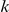 represents different replications of the data for given individual and experimental condition. The intrinsic phenotype 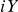 and experimental conditions are treated as random effects and covariates 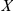
's are treated as fixed effects. Extension to generalized linear model is straightforward.

### Hapmap cell lines and intrinsic growth

HapMap cell lines were purchased from the non-profit Coriell Institute for Medical Research (http://www.coriell.org/) and growth rates were measured using alamarBlue assay as described in Stark et al. [12].

The alamarBlue assay gives a measure of the number of proliferating cells 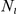 at time 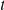:
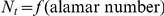
where 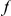 is some increasing function. Thus growth rate can in principle be computed as
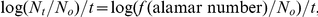
which is also some function of the alamar number. With a slight abuse of notation, we will refer to this alamar number as the growth rate. It should be noted that the approach we describe here holds generally regardless of the assay used to measure the number of proliferating cells.

We use MEM to compute an intrinsic growth rate for each cell with the following model:
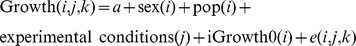
where the index 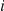 identifies the cell line, pop is the population to which the cell line belongs to (CEU,YRI, ASW, or ASN), iGrowth0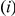 represents the intrinsic growth rate of the cell line, experimental conditions can represent a large range of different experimental conditions, and 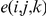 is an error term. The index 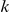 represents different replications of the data for given individual and experimental condition. We set the gender and population as fixed effects and experimental conditions and iGrowth0 as random effects.

The term iGrowth0 is a cell line specific intercept (so it is different for each cell line) and can be interpreted as (some monotone function of) the intrinsic growth rate of each individual. This term is (by construction) orthogonal to gender and population. In general, it may make more sense to include the population and gender effect in the intrinsic growth rate so we define iGrowth as the sum of the iGrowth0 and the estimated effects of population and gender.
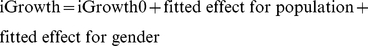
The term “experimental conditions” could be allowed to be more than one-dimensional. For our dataset it was sufficient to use “technician” as the experimental condition. The reason for this choice was that each technician's work was for the most part concentrated at roughly the same time (within 6 months) so the experimental conditions such as thaw history are likely to be reasonably homogeneous. Our results were robust to using other combinations of experimental conditions such as a combination of technician, drug and population. We found no need to account for the trio structure since the correlation coefficient between parent and child was not significantly different from zero. This fact should not be interpreted as lack of heritability but that the level of noise was too high to be able to estimate the correlation with the given sample size.

Growth rate itself was quite normally distributed. The additivity assumption of the model may be better achieved in the log scale but we did not notice much difference in the overall results when we tried different transformations so we used the untransformed variable. We fit the mixed effects model using the lme4 [45] package for the R Statistical Software [46]. P-values for the fixed effects were calculated using likelihood ratio tests after fitting the models with maximum likelihood option (REML = FALSE).

Gene expression data was generated by our lab for phase I/II CEU and YRI cell lines using Affymetrix GeneChip Human Exon 1.0 ST array as described by Zhang et al. [27]. Association between gene expression and growth rate was calculated using a linear model with log-transformed gene expression data as response and intrinsic growth, population and gender as covariates.
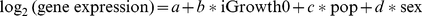
Surrogate Variable adjustment was done using the SVA package [28], [47]. FDR was computed using Storey's qvalue package[48], [49]. Figures were generated using the graphic capabilities of R and the ggplot2 package [50] in R. Functional term enrichment was assessed using DAVID [29], [30]. Genes associated with intrinsic growth at 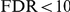
% were used as significant genes and the default Homo Sapiens list was used as background.

Genome wide association between genotype and intrinsic growth was performed using the PLINK v1.97 software [51] (http://pngu.mgh.harvard.edu/purcell/plink/). CEU (I/II and II) and YRI (I/II and III) unrelated cell lines were used with draft release 2 consensus genotype (which passed QC across all 11 populations from HapMap 3 samples) downloaded from the HapMap Project website.

### Enrichment of growth associated genes among targets of growth associated SNPs

We use three methods to assess the enrichment of growth associated genes among targets of growth associated SNPs.

First we use the hypergeometric distribution and tests (Fisher's test) whether the overlap is more significant than one would get with a random set of genes. This method computes the exact p-value but assumes independence between genes.

The second method accounts for the correlation structure between target genes by simulation, which will induce a correlation structure between simulated genes similar to the observed one. For this purpose, we permute the phenotype 1000 times and for each permutation we perform GWAS, select the top SNPs (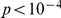), query the target genes for the top SNPs (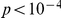) using SCANdb, and calculate the Fisher's test p-value for the overlap between growth genes and target genes. Finally, we compute an empirical p-value for the enrichment as the proportion of times the simulated Fisher's p-value was smaller than the observed Fisher's p-value.

The third method accounts for the fact that the data from the same individuals are used when computing the intrinsic growth-gene association as well as the intrinsic growth-SNP association. The simulated target genes are generated as described in the second method. The intrinsic growth associated genes are generated using the same permuted phenotype used to generate the target genes (FDR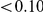). The association p-value is computed by regressing the simulated intrinsic growth on gene expressions (without expression heterogeneity adjustment, 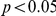). For each simulation, the Fisher's p-value is computed for the overlap between simulated target genes and simulated intrinsic growth associated genes. Finally, we compute an empirical p-value for the enrichment as the proportion of times the simulated p-value was smaller than the observed p-value.

## Supporting Information

Table S1 Intrinsic Growth. This file lists the full list of intrinsic growth rate computed using MEM.
(CSV)

Table S2 Growth-associated genes. This file lists the p-values and effect sizes of the association between intrinsic growth rate and gene levels. Both the SVA-adjusted and unadjusted p-values are included.
(TXT)

Table S3 KEGG pathways enriched in growth-associated gene set. This table shows the top KEGG pathways enriched in our growth-associated gene set. It was obtained using the DAVID Bioinformatic Resources.
(PDF)
