# Supplementary material for: Mixed Effects Modeling of Proliferation Rates in Cell-Based Models: Consequence for Pharmacogenomics and Cancer
Source: PLoS Genet. 2012 Feb 9;8(2):e1002525. doi: 10.1371/journal.pgen.1002525 (PMC3276560; doi:10.1371/journal.pgen.1002525)
Supplement: Table S3 — KEGG pathways enriched in growth-associated gene set. This table shows the top KEGG pathways enriched in our growth-associated gene set. It was obtained using the DAVID Bioinformatic Resources. (PDF) [file pgen.1002525.s003.pdf]

Term	Count	%	PValue	Bonferroni	Benjamini
hsa04142:Lysosome	39	1.40	3.56E-05	6.77E-03	6.77E-03
hsa00970:Aminoacyl-tRNA biosynthesis	18	0.64	2.15E-04	4.03E-02	2.04E-02
hsa04150:mTOR signaling pathway	20	0.72	6.41E-04	1.15E-01	4.00E-02
hsa04115:p53 signaling pathway	24	0.86	6.45E-04	1.16E-01	3.04E-02
hsa05120:Epithelial cell signaling in Helicobacter pylori infection	23	0.82	1.63E-03	2.68E-01	6.06E-02
hsa04910:Insulin signaling pathway	38	1.36	1.85E-03	2.98E-01	5.74E-02
hsa04120:Ubiquitin mediated proteolysis	38	1.36	2.46E-03	3.75E-01	6.49E-02
hsa04662:B cell receptor signaling pathway	24	0.86	2.85E-03	4.20E-01	6.58E-02
hsa05215:Prostate cancer	27	0.97	3.32E-03	4.70E-01	6.82E-02
hsa04620:Toll-like receptor signaling pathway	29	1.04	5.35E-03	6.41E-01	9.73E-02
hsa04010:MAPK signaling pathway	63	2.25	6.17E-03	6.93E-01	1.02E-01
hsa05221:Acute myeloid leukemia	19	0.68	6.86E-03	7.32E-01	1.04E-01
hsa04144:Endocytosis	46	1.65	7.07E-03	7.42E-01	9.90E-02
hsa05212:Pancreatic cancer	22	0.79	8.06E-03	7.87E-01	1.04E-01
hsa04722:Neurotrophin signaling pathway	32	1.14	1.69E-02	9.62E-01	1.96E-01
hsa04110:Cell cycle	32	1.14	1.90E-02	9.74E-01	2.04E-01
hsa04210:Apoptosis	24	0.86	1.95E-02	9.77E-01	1.99E-01
